# Effect of the Free Volume on the Electronic Structure of Cu_70_Zr_30_ Metallic Glasses

**DOI:** 10.3390/ma13214911

**Published:** 2020-10-31

**Authors:** Simon Evertz, Jochen M. Schneider

**Affiliations:** Materials Chemistry, RWTH Aachen University, Kopernikusstr 10, D-52074 Aachen, Germany; schneider@mch.rwth-aachen.de

**Keywords:** metallic glass, free volume, electronic structure, ab initio

## Abstract

While it is accepted that the plastic behavior of metallic glasses is affected by their free volume content, the effect on chemical bonding has not been investigated systematically. According to electronic structure analysis, the overall bond strength is not significantly affected by the free volume content. However, with an increasing free volume content, the average coordination number decreases. Furthermore, the volume fraction of regions containing atoms with a lower coordination number increases. As the local bonding character changes from bonding to anti-bonding with a decreasing coordination number, bonding is weakened in the volume fraction of a lower coordination number. During deformation, the number of strong, short-distance bonds decreases more for free volume-containing samples than for samples without free volume, resulting in additional bond weakening. Therefore, we show that the introduction of free volume causes the formation of volume fractions of a lower coordination number, resulting in weaker bonding, and propose that this is the electronic structure origin of the enhanced plastic behavior reported for glasses containing free volume.

## 1. Introduction

Plastic deformability is crucial for structural applications of metallic glasses [1]. Increasing the free volume content has been proposed to enhance the plastic deformation of metallic glasses and is often referred to as “structural rejuvenation” [2]. Recently, an enhanced free volume content was reported to induce work hardening and hence enable stable plastic deformation [3].

Free volume is inherent to the glassy state, as its density varies depending on the synthesis conditions [2], and consists of volume fractions containing atoms with a lower coordination number [4], allowing the atoms to move within their nearest neighbor cage without energy change [5]. Therefore, the atomic mobility is enhanced [4,6] as the free volume lowers the energy barrier for shear transformations in metallic glasses [4,6], promoting plastic deformability [7]. While the literature often focusses on free volume-induced changes in internal energy [2,7,8,9,10] and topology [3,11,12,13], the effect of free volume on the electronic structure has been overlooked thus far. Cu_70_Zr_30_ metallic glasses have previously been predicted to be brittle based on ab initio methods [14,15]. Therefore, the goal of this study is to understand the effect of free volume on the electronic structure and hence chemical bonding based on ab initio calculations by systematically increasing the free volume content of Cu_70_Zr_30_.

## 2. Methods

Density-functional-theory (DFT)-based [16] ab initio molecular dynamics calculations were carried out in this work. To create glassy structural models, the modeling routine introduced by Hostert et al. [17] was employed. The initial supercell contained 115 atoms, which were initially randomly distributed on a bcc-lattice (115 atoms and 13 vacancies) [17]. This supercell was heated up with a timestep of 1 fs to 4000 K for 400 fs in a canonical ensemble by the scaling of velocities and subsequently quenched to 0 K by geometry relaxation employing the openMX 3.9.2 code [18,19]. Electronic potentials with the general gradient approximation [20] and the basic functions Cu6.0S-s3p3d3 and Zr7.0-s3p3d3f1 were applied, where the first symbol designated the chemical element and cutoff-radius and the last set of symbols defined the primitive orbitals. A 1 × 1 × 1 k-grid and an energy cutoff-radius of 150 Ry were used. After quenching to 0 K, the volume was relaxed by employing the Vienna Ab initio Simulation Package (VASP) [21,22] with projector-augmented wave potentials [22,23] using the Perdew–Burke–Ernzerhof functional [20]. Integration over the Brillouin zone was conducted on a 3 × 3 × 3 Monkhorst–Pack k-grid [24]. This heating-quenching-relaxation cycle described above was repeated until the volume change between two subsequent cycles was smaller than 2%.

To create different free volume contents, up to five atoms were removed from the amorphous structural model while keeping the composition approximately constant. To avoid a vacancy-like atomic configuration around the position of the removed atom, the supercell was heated to 4000 K for 400 fs by velocity scaling in a canonical ensemble and subsequently quenched to 0 K by geometry relaxation. This allowed the free volume to distribute within the supercell. To probe the significance and reproducibility of the observed results, four independent structural models were developed from the initial crystalline supercell up to the free volume-containing supercells.

The bulk modulus was calculated from the ground state by fitting the energy-volume data with the Birch–Munarghan equation of state [25], and the shear modulus was calculated using volume-conserving distortions [26]. To calculate pair distribution functions, taking the atomic scattering factors into account [17], the supercell was heated to 300 K for 300 fs. The pair distribution functions were averaged over the last 200 fs. For bonding analysis, the crystal orbital Hamilton populations (COHPs) [27] were obtained from the LOBSTER code (version 3.2.0) [28,29,30].

## 3. Results and Discussion

**Figure 1:**
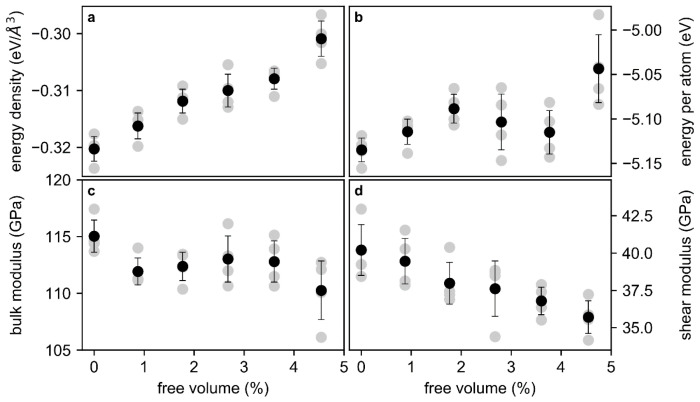
Ab initio total energy density (**a**), energy per atom (**b**), bulk modulus (**c**), and shear modulus (**d**) of Cu_70_Zr_30_. Gray symbols mark the result of a single calculation and black symbols mark the average of the data points per free volume content, while the variability bars represent plus and minus one standard deviation of the average value of the properties listed in Table 1, which were obtained for the different structural models investigated in the study.

Before the electronic structure and topology are analysed, the changes of total energy and elastic moduli as a function of increasing free volume content are investigated and are shown in Figure 1. The variability range indicated does not represent the numerical error of the calculation, but the variability of plus and minus one standard deviation of the average of the properties listed in Table 1, which were obtained for the different structural models investigated in the study.

The free volume is the excess mean atomic volume compared to the reference structural model that is based on a supercell containing 115 atoms, which has a free volume content of 0% per definition. The internal energy of the glass is represented here as the energy density and energy per atom (Figure 1a,b). The energy density is used to normalize the total energy of the structural models with the same free volume content, as the absolute volumes of the structural models are not identical. With the free volume content increasing by 4.6%, the energy density increased by 6.4% and the energy per atom by 1.8%. This increase of energy is larger than the variability between the structural models and is consistent with the literature [2,31]. However, the internal energy at free volume contents of 2.7 and 3.6% deviates to lower energies from the increasing internal energy observed before.

The bulk modulus (Figure 1c) varies from 110 to 115 GPa and hence by 4.3%. This small change of the bulk modulus with an increasing free volume content reflects bond weakening. The shear modulus (Figure 1d) decreases linearly by 10% with an increasing free volume content. Therefore, the energy barrier for shear transformations decreases [32], which is consistent with the increased internal energy [8]. The effect of the free volume on the resistance to hydrostatic deformation is small but significant.

**Figure 2:**
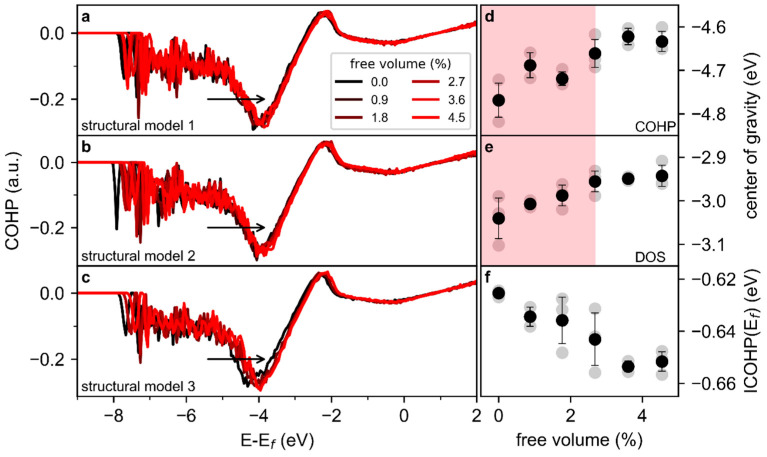
Global bonding analysis of free volume-containing Cu_70_Zr_30_. Crystal orbital Hamilton population (COHP) of three Cu_70_Zr_30_ metallic glass structural models (**a**–**c**). Free volume-induced changes in the integrated COHP up to the Fermi level, in the center of gravity of the COHP, and in the density of states are depicted in (**d**–**f**), respectively. In (**d**–**f**), black symbols indicate the average of the quantities based on all structural models, and gray symbols indicate the values of the individual calculations. The variability bars indicate the range of plus and minus one standard deviation from the average value obtained for the different structural models investigated in this study.

To reveal the effect of an increasing free volume on the electronic structure, partitioning of the bond energy is analysed: The COHPs of the three structural models, which are based on different initial configurations, are similar (Figure 2a–c), showing bonding contributions below approx. −2.5 eV and anti-bonding contributions close to the Fermi level. However, it is evident that with an increasing free volume content, the COHPs are shifting towards the Fermi level, which is indicated by the arrows in Figure 2a–c and is further visualized by the free volume-induced changes of the center of gravity (CoG) of the COHP and the electronic density of states (DOS, Figure S1) depicted in Figure 2d,e, as calculated by Equation (1).

(1) $$CoG_{X}=\;\frac{\int^{}\left(X\cdot E\right)\;dE}{\int^{}X\;dE}$$

Here, *X* is the COHP or DOS, respectively, and *E* is the energy of the electronic states. For free volume contents up to 2.7%, the *CoG* shift towards the Fermi level is larger than the variation of data at a constant free volume (red shaded area in Figure 2d,e). However, for free volume contents larger than 2.7%, the shift of the *CoG* is smaller than the variation at a constant free volume and hence, the *CoG* remains constant with respect to the Fermi level. This is in line with the critical free volume content for yielding of metallic glasses proposed by Wang et al. [33].

The integrated COHP (ICOHP) at the Fermi level, which is a measure of the bond strength [29], exhibits a subtle decreasing trend and strongly dispersed data (Figure 2f). The difference in ICOHP between the minimum and maximum free volume contents (0.0 and 4.6%) is −0.3 meV/atom. Due to the numerical precision of ab initio calculations [34] and the orthogonal projection of the electrons [29], this difference is appraised as not significant, while the scattering within the set of calculations analysed here indicates a slightly decreasing ICOHP with an increasing free volume content. Therefore, while the electrons occupy higher energetic electronic states with an increased free volume content, the analysis of the total COHP confirms that the effect of the free volume on the overall bond strength is small but significant.

**Figure 3:**
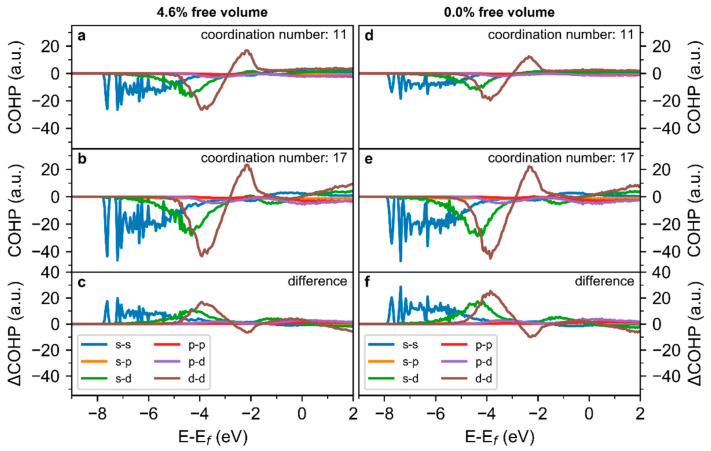
Band-resolved COHP of Cu_70_Zr_30_ metallic glass with a (**a**–**c**) 4.6% and (**d**–**f**) 0.0% free volume content. (**a**,**d**) show the COHP for atoms with a coordination number of 11, (**b**,**e**) for a coordination number of 17, and (**c**,**f**) the difference between a low and high coordination number.

To investigate the influence of the free volume on directional and non-directional bonds spatially resolved, the COHPs are separated into the contributions of the electronic bands of the Cu_70_Zr_30_ samples with free volume contents of 4.6% (Figure 3a–c) and 0.0% (Figure 3d–f). As the total COHPs of the structural models are consistent (Figure 2a–c) and due to computational constraints, the band-resolved analysis has been conducted only for structural model 1. Based on the electronic band contributions (Figure 3a,b,d,e), the interactions between the s-bands (s-s), the s- and d-bands (s-d), and the d-bands (d-d) are dominating the bonding in Cu_70_Zr_30_. As observed for the total COHPs (Figure 2a–c), only minor differences exist between the COHPs for the same structural model but different free volume contents. Therefore, both directional (s-d and d-d bonds) and non-directional bonding (s-s [35]) is affected in a similar way by the free volume. However, the COHPs for low coordination numbers exhibit less bonding states below −2.5 eV, as well as an enhanced number of anti-bonding states between −1.5 eV and the Fermi level (Figure 3c,f). As this analysis indicates that the local atomic structure predominantly affects the bonding, the effect of the free volume on the local bonding is probed next.

**Figure 4:**
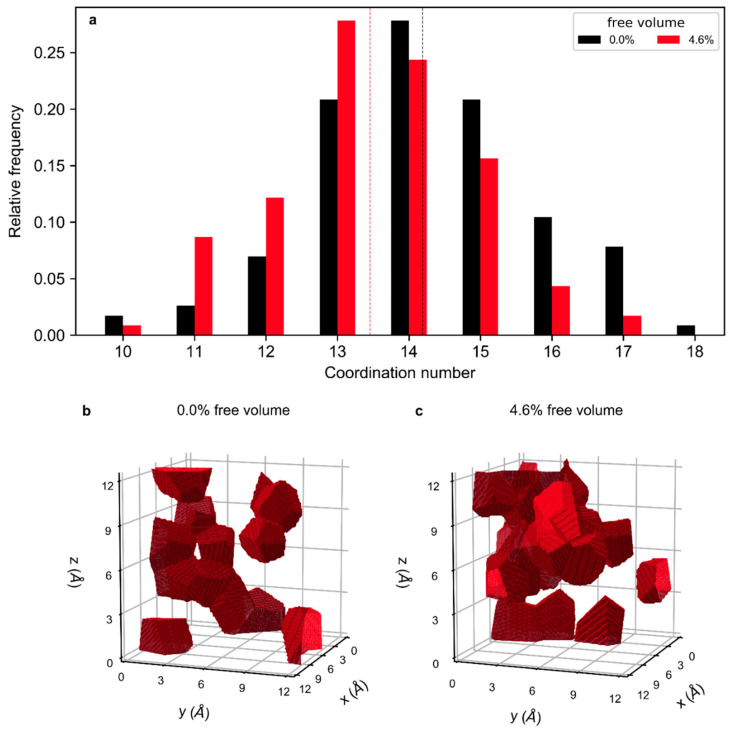
Global and spatially resolved coordination number analysis. (**a**) Relative frequency of occurrence of a coordination number in the sample. Black bars represent the coordination number of a sample with a 0.0% free volume content, and red bars of a sample with a 4.6% free volume content. Dashed lines mark the respective average coordination number. Iso-configuration surfaces enclosing regions with a coordination number of 12 and lower in the simulation cell for a glass with a 0.0% and 4.6% free volume are shown in (**b**,**c**), respectively.

To this end, the distribution of coordination numbers, i.e., the number of neighbors of an atom within the first coordination shell, which is discussed later in Figure 6, is analysed by comparing the simulation cell containing no free volume with the one containing a 4.6% free volume, as presented in Figure 4a. The range of coordination numbers observed is consistent with the literature [36]. The average coordination number decreases from 14.2 to 13.5 as free volume is introduced. To visualize the impact of the free volume on the spatial distribution of coordination numbers, iso-coordination surfaces that enclose volume sections containing atoms with a coordination number ≤ 12 are shown in the simulation cell in Figure 4b,c. In the configuration without free volume (Figure 4b), these iso-coordination sections are partly interconnected and populate 11.3% of the volume of the supercell. With an increased free volume content of 4.6% (Figure 4c), the volume fraction of the iso-coordination sections increases by 11.8%. Therefore, the introduction of a 4.6% free volume causes the volume fraction containing atoms with coordination numbers ≤ 12 to increase to 23.1%.

**Figure 5:**
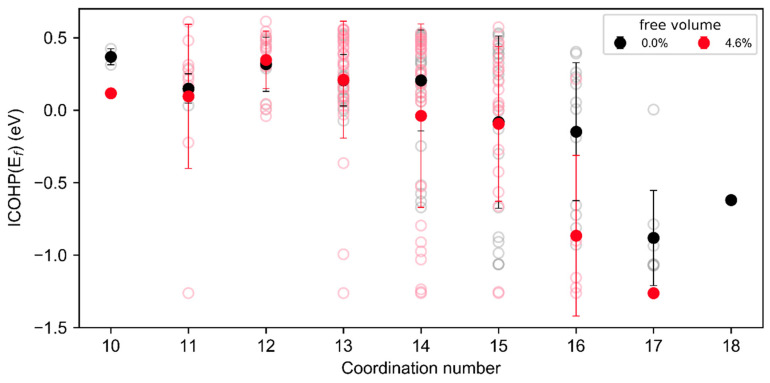
Integrated COHP (ICOHP)(E_f_) as a function of the atomic coordination number. Full points mark the mean ICOHP at each coordination number and variability bars indicate plus and minus one standard deviation from the average of all bonds with the respective coordination number. Open symbols mark the ICOHP(E_f_) of the individual atoms considered.

The analysis of the ICOHP(E_f_) of individual atoms as a function of the coordination number (Figure 5) emphasizes the bond weakening in regions of a low coordination number: Atoms with a coordination number ≤ 14 exhibit a positive ICOHP(E_f_) and hence overall anti-bonding interactions. Therefore, the bonding in the volume fraction with a coordination number ≤ 14 is weakened. For more densely packed atoms with a coordination number > 14 and larger, the ICOHP(E_f_) is negative. Hence, interactions with the nearest neighbors for atoms with a coordination number > 14 are bonding strongly. The magnitude of the bond energy range for coordination numbers between 14 and 17 may originate from the inherent structural heterogeneities of metallic glasses [6]. From Figure 5, it is clearly visible that the ICOHP(E_f_) as a function of the coordination number is independent on the overall free volume content in the sample. However, it is also evident that the number of weakened bonds clearly increases due to the shift of the coordination number distribution to lower values and the increasing volume fraction of regions containing atoms with a lower coordination number. This is consistent with the reduced shear modulus (Figure 1), and the reported lowered activation energy for shear transformations [4,6].

**Figure 6:**
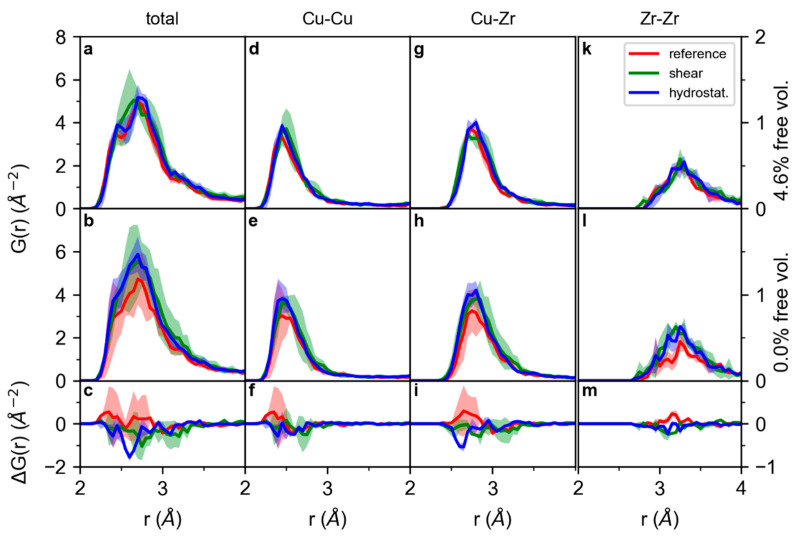
Total pair distribution functions of Cu_70_Zr_30_ of the undeformed, 2% sheared, and 2% hydrostatically enlarged cells for a sample containing a 4.6% free volume (**a**) and 0.0% free volume (**b**) and the difference in the pair distribution functions with a 4.6% and 0.0% free volume (**c**). (**d**–**f**) present the corresponding partial pair distribution functions for Cu-Cu, (**g**–**i**) Cu-Zr, and (**k**–**m**) Zr-Zr bonds. The pair distribution functions (PDFs) shown are the average of three independently calculated samples to enlarge the overall sample size. Shaded areas represent the variability obtained for the different structural models investigated in this study.

To analyse the effect of the free volume on the short-range order, the pair distribution function (PDF) of an undeformed, a 2% sheared, and a 2% hydrostatically expanded glass with a 4.6% free volume content (Figure 6a) and 0% free volume (Figure 6b) are compared by showing the difference in the PDF in Figure 6c. To enlarge the overall sample size, the average of three independently calculated configurations is shown in Figure 6. The complete pair distribution functions presented in Figure S2 clearly show the lack of long-range order in the samples. The bond distances of 2.5, 2.75, and 3.2 Å for Cu-Cu, Cu-Zr, and Zr-Zr, respectively, are consistent with the literature [36,37] within 0.15 Å, which has been reported for ab initio structural models of metallic glasses before [38]. However, as the deviation from the literature is consistent throughout the structural models investigated and this analysis is based on a comparison with the structural model without free volume as a reference, the conclusions from this study are considered valid. While the bond distribution is similar for deformed and undeformed, free-volume-containing and free-volume-free samples, small changes can be observed in the difference curve (Figure 6c): With an increased free volume content, the number of bonds is reduced for the sheared and hydrostatically deformed cells. While this effect is small, the variability bands of the PDF of the sheared and hydrostatically deformed samples do not overlap completely with the variability band of the reference PDF, indicating a decreased number of bonds. Therefore, the effect of the free volume content on the number of bonds is small but significant.

By not only comparing the total PDFs, but also focusing on the partial Cu-Cu PDFs (Figure 6d–f), Cu-Zr PDFs, (Figure 6g–i) and Zr-Zr PDFs (Figure 6k–m), the bond depletion discussed for the total PDF can be observed in the partial Cu-Cu and Cu-Zr PDFs, which are the main contributors to the total PDF: While the deformation-induced bond depletion with an increased free volume content is not significant for Cu-Cu bonds (Figure 6f), the number of Cu-Zr bonds in the hydrostatically deformed samples is reduced on the short bond-distance side of the first peak of the PDF for the free volume-containing glass compared to the glass without free volume. However, for the undeformed cell, a larger peak, i.e., a larger number of bonds, is observed for Cu-Cu and Cu-Zr in the free volume-containing cell (Figure 6f,i), while during deformation, these additional bonds are no longer present. For the Zr-Zr bonds (Figure 6k–m), a small increase in the number of bonds with free volume content in the undeformed cell is observed, while the effect of deformation on the number of bonds for the free volume-containing system compared to the system without free volume is minor.

Hence, small topological differences between 0.0 and 4.6% free volume-containing samples are observed: The number of bonds in the short-range order increases with the free volume content for undeformed systems, while for shear and hydrostatic deformation, the number of short-distance and hence strong bonds in the short-range order is reduced more for free volume-containing systems than for systems without free volume. This originates from a deformation-induced depletion in the number of Cu-Zr bonds. The observed changes in topology are consistent with the weaker bonding under shear deformation proposed based on the electronic structure analysis and the reported decreased barrier for shear transformations [4,5].

## 4. Conclusions

A systematic, ab initio calculation-based comparison pf Cu_70_Zr_30_ metallic glasses with free volume contents varying from 0.0% to 4.6% revealed that the overall bond strength based on the integrated COHP changes marginally with an increasing free volume content, while the populated electronic states and bond energy contributions shift by 0.1 eV to higher energies. However, the average coordination number decreases from 14.2 to 13.5 with an increasing free volume content from 0.0 to 4.6%, since the volume fraction of regions containing atoms with a coordination number ≤ 12 increases significantly: For samples without free volume, the volume fraction of regions containing atoms with a coordination number ≤ 12 occupies 11.3% of the simulation cell volume, while this volume fraction increases to 23.1% for glasses with a 4.6% free volume content. Due to an increasing number of anti-bonding contributions to the local bonding with a decreasing coordination number, the ICOHP(E_f_) exhibits positive bonds of atoms with coordination numbers ≤ 14, indicating the dominance of anti-bonding contributions. Therefore, the bonding is weakened locally in the volume fraction of lower coordination. Topology-wise, under shear and hydrostatic deformation, free volume-containing samples show a larger decrease in strong, short-distance bonds with an increased free volume than samples without free volume, indicating additional bond weakening. Therefore, the introduction of free volume causes the formation of volume fractions of lower coordination. Due to a local rise of anti-bonding states in these volume fractions of lower coordination, bonding is weakened. We propose that this local bond weakening is the electronic structure origin of the enhanced plastic behavior reported for free volume-containing metallic glasses.

## Figures and Tables

**Table 1:** Free volume, total energy, energy per atom, supercell volume, energy density, and bulk and shear modulus of the four structural models developed in this study.

Free Volume (%)	Total Energy (eV)	Energy per Atom (eV/Atom)	Supercell Volume (Å^3^)	Energy Density (eV/Å^3^)	Bulk Modulus (GPa)	Shear Modulus (GPa)
**Structural model 1**						
0.0	−590.6	−5.12	1847.5	−0.320	114.4	39.2
0.9	−582.2	−5.11	1847.5	−0.315	111.3	37.9
1.8	−576.3	−5.10	1847.5	−0.312	113.4	37.2
2.7	−576.4	−5.14	1847.5	−0.312	116.1	38.7
3.6	−566.4	−5.10	1847.5	−0.307	111.5	35.5
4.5	−548.1	−4.98	1847.5	−0.297	106.1	35.9
**Structural model 2**						
0.0	−589.9	−5.13	1857.0	−0.317	113.7	40.2
0.9	−582.5	−5.11	1857.0	−0.314	111.3	38.1
1.8	−574.2	−5.08	1857.0	−0.309	112.3	36.9
2.7	−567.3	−5.06	1857.0	−0.305	112.0	34.4
3.6	−570.9	−5.14	1857.0	−0.307	115.1	37.4
4.5	−557.3	−5.06	1857.0	−0.300	112.1	34.2
**Structural model 3**						
0.0	−592.9	−5.16	1831.8	−0.324	114.9	43.0
0.9	−585.8	−5.14	1831.8	−0.320	111.1	41.5
1.8	−577.1	−5.11	1831.8	−0.315	108.3	40.4
2.7	−573.2	−5.12	1831.8	−0.313	103.7	38.5
3.6	−569.8	−5.13	1831.8	−0.311	102.3	37.9
4.5	−559.2	−5.08	1831.8	−0.305	96.5	35.6
**Structural model 4**						
0.0	−588.7	−5.12	1839.0	−0.320	114.6	38.4
0.9	−581.7	−5.10	1839.0	−0.316	111.2	40.3
1.8	−572.4	−5.07	1839.0	−0.311	110.4	37.4
2.7	−569.4	−5.08	1839.0	−0.309	110.7	38.8
3.6	−564.0	−5.08	1839.0	−0.307	110.6	36.4
4.5	−554.6	−5.04	1839.0	−0.302	110.1	37.2

## Supplementary Materials

The following are available online at https://www.mdpi.com/1996-1944/13/21/4911/s1: Figure S1: Density of states of Cu_70_Zr_30_ metallic glasses with different free volume contents. Figure S2: Pair distribution functions of Cu70Zr30 structural models containing different amounts of free volume.
