# Supplementary material for: Effect of the Free Volume on the Electronic Structure of Cu70Zr30 Metallic Glasses
Source: Materials (Basel). 2020 Oct 31;13(21):4911. doi: 10.3390/ma13214911 (PMC7672583; doi:10.3390/ma13214911)
Supplement: Supplementary file 1 [file materials-13-04911-s001.docx]

Supplementary Information

Effect of free volume on the electronic structure of Cu_70_Zr_30_ metallic glasses

S. Evertz and J. M. Schneider


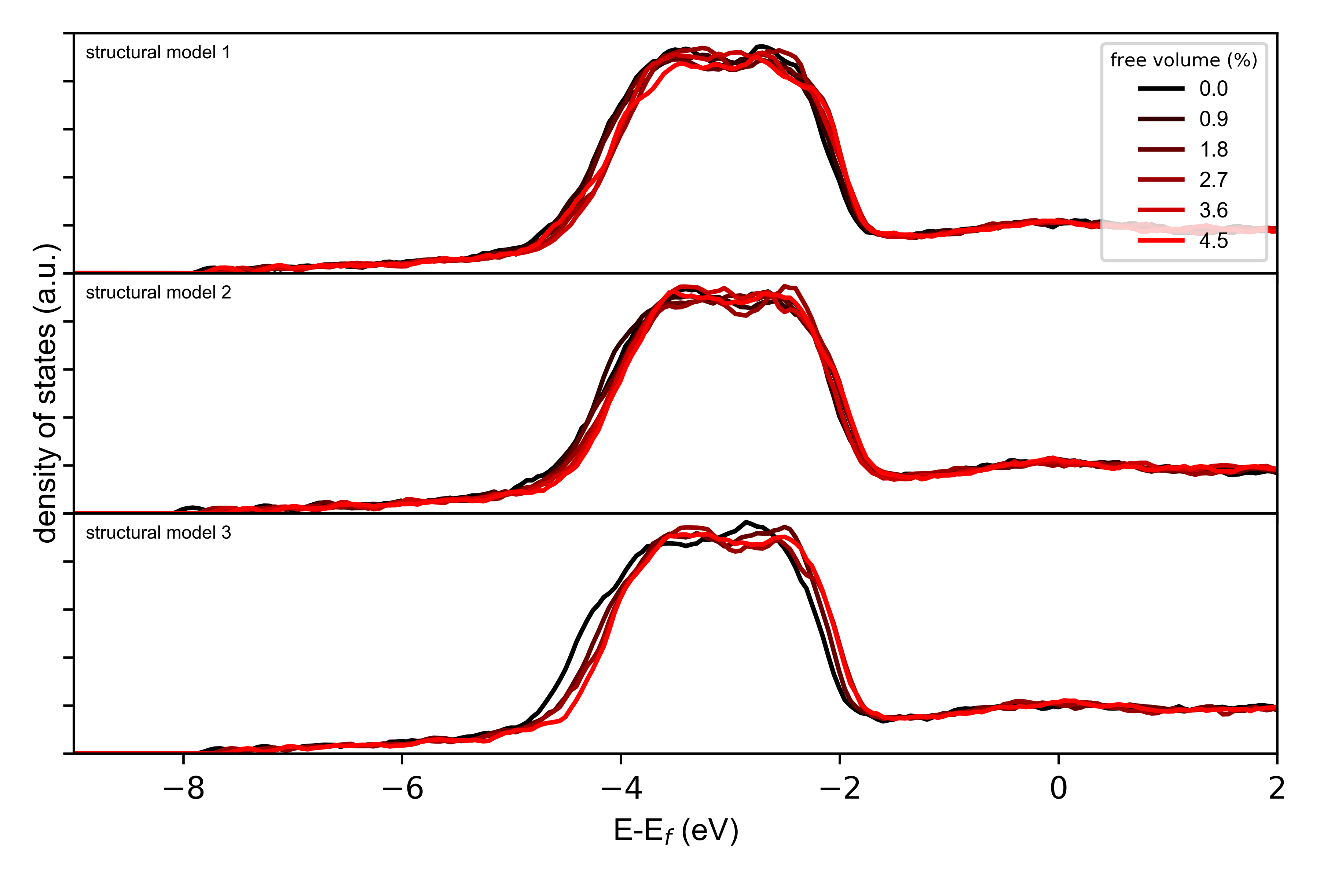


Figure S1: Density of states of Cu_70_Zr_30_ metallic glasses with different free volume contents


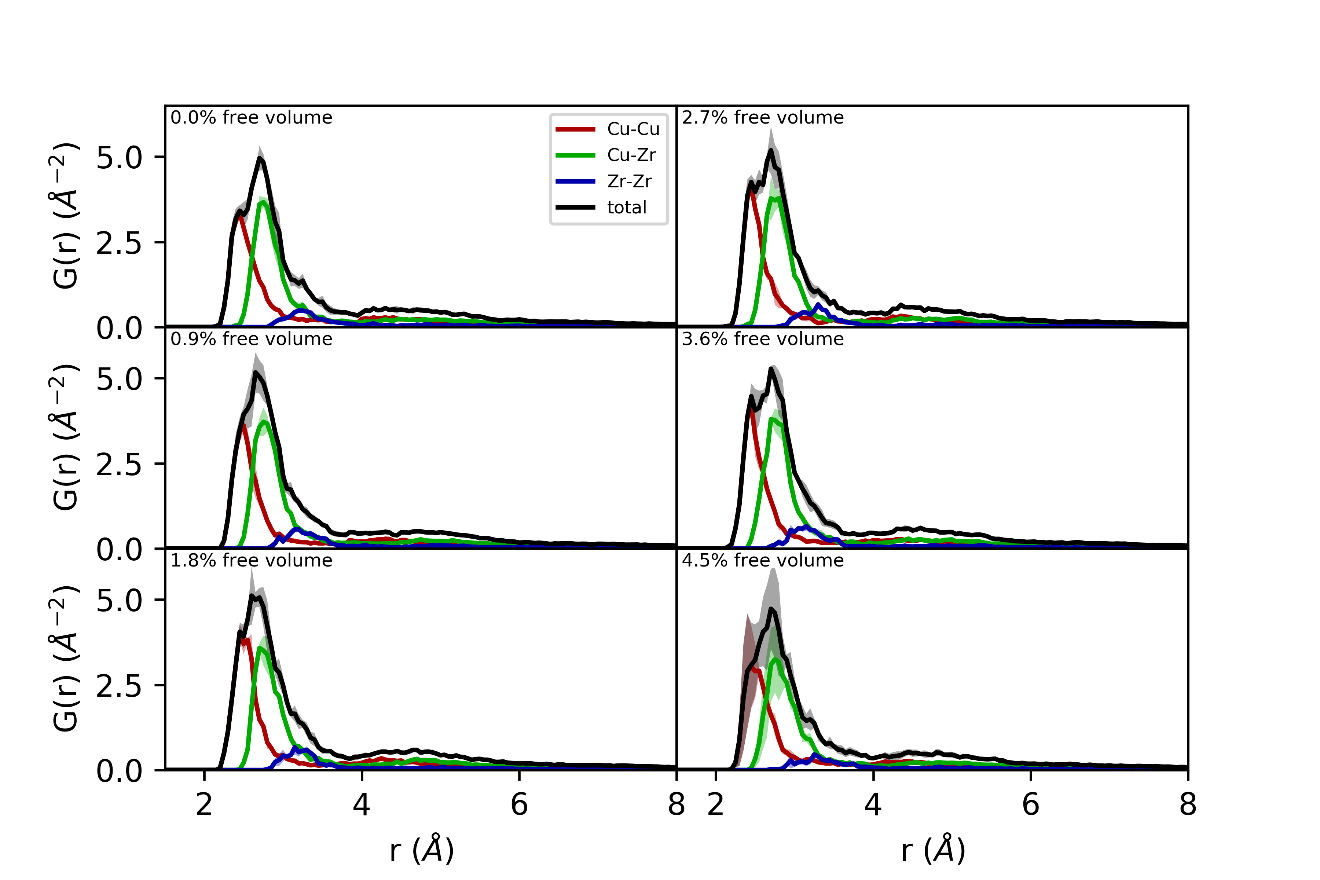


Fig. S2: Pair distribution functions of Cu_70_Zr_30_ structural models containing different amounts of free volume.
